# Effect of target thickness and laser irradiance on the back-reflection-enhanced laser-induced breakdown spectroscopy signal in glass

**DOI:** 10.1038/s41598-023-34227-3

**Published:** 2023-05-03

**Authors:** Asmaa Elhassan, Mohamed Abdel-Harith, Mahmoud Abdelhamid

**Affiliations:** 1 Higher Technological Institute (HTI), 10th of Ramadan City, 6th of October Branch, Giza, Egypt; 2 National Institute of Laser Enhanced Sciences (NILES), Cairo University, Giza, Egypt

**Keywords:** Applied physics, Techniques and instrumentation

## Abstract

In the work that is being presented here, the effect of sample thickness and laser irradiance on the reduction of the signal-to-background ratio SBG and the plasma parameters, specifically electron temperature and electron density, is being investigated using back-reflection-enhanced laser-induced breakdown spectroscopy (BRELIBS). Copper and silver discs that had been highly polished were attached to the back surface of the glass target, and the Nd-YAG laser beam that was focused on the front surface of the target was tuned to its fundamental wavelength. The thicknesses of the transparent glass samples that were analysed were 1 mm, 3 mm, and 6 mm. One is able to achieve a range of different laser irradiance levels by adjusting the working distance that exists between the target sample and the focusing lens. The end result of this is that the signal-to-background ratio in the BRELIBS spectra of thicker glass samples is significantly lower as compared to the ratio in the spectra of thinner glass samples. In addition, a significant influence of modifying the laser irradiance (by increasing the working distance on the SBG ratio) is seen at various glass thicknesses for both BRELIBS and LIBS, with BRELIBS having a better SBG. Nevertheless, the laser-induced plasma parameter known as the electron temperature has not been significantly impacted by the decrease in the glass thickness.

## Introduction

Laser-Induced Breakdown Spectroscopy (LIBS) is a spectrochemical analytical technique used for the elemental analysis of any target material, whether gas, liquid, or solid^1–3^. Since the early emergence of LIBS in 1982, the technique has been applied in numerous fields, industrial^4–6^, biology^7^, medicine^8^, environmental^9,10^, forensic^11,12^, archeological^13,14^, geology^15,16^, etc. Details of the fundamentals and applications of LIBS can be found in many published books^17,18^ and review papers^19–21^. Focusing a nanosecond, picosecond, or femtosecond laser pulses of a few mJ energy per pulse onto the surface of a solid target leads to melting, boiling, and evaporation of a tiny amount of the target material. The rise of the vapor temperature due to heat absorption from the laser pulse dissociates the molecules into atoms. With further increase in the temperature (6000–60,000 K), atoms are ionized, and a plasma plume consisting of a collection of ions and swirling electrons is evolved at such elevated temperature^22,23^. As the plasma plume cools down, it gets rid of the previously absorbed energy by emitting photons of light. The emitted plasma light is collected, dispersed, and displayed as an emission spectrum. Qualitatively, the spectral lines in the obtained spectrum characterize the elements in the plasma and, consequently, the target material. On the other hand, quantitatively, there is a proportionality relation between the spectral lines’ intensity and the concentration of the corresponding elements, considering the self-absorption and the matrix effects.

Two critical parameters that characterize the expanding laser-induced plasma plume are the plasma temperature and the electron density. The widespread of LIBS worldwide refers to its appealing pros, such as its simplicity, cost-effectiveness, no or minimal preparation of the target material, quasi-nondestructive, noninvasive, and can detect low and high atomic number elements simultaneously. However, LIBS has a significant limitation in its relatively inferior limit-of detection compared to other conventional analytical techniques, such as ICP, LA-ICP, and EDX. Promoting LIBS sensitivity has represented an important challenge for researchers during the last two decades. Different routes have been followed and suggested in this concern. For example, ultrashort laser pulses were useful but relatively expensive compared to nanosecond laser pulses. Double pulse LIBS provided a good step forward but raised the cost and complicated the setup. Nano-enhanced LIBS was an excellent solution that improved the LIBS sensitivity effectively. However, in most of these suggested amendments of the LIBS arrangements, excluding the NELIBS, the technique’s setup is becoming more complicated and costly. In addition, contrary to the standard LIBS, in situ measurements are often unavailable utilizing the newly suggested setups. Recently the technique, known as back-reflection-laser-induced breakdown spectroscopy (BRELIBS) applying LIBS onto transparent solid targets^24^, is straightforward and inexpensive and does not need any amendments or additional equipment to the experimental setup, besides keeping the same main pros of the conventional LIBS. Briefly, in BRELIBS, a metallic reflector in direct contact with the transparent target’s back surface is utilized to strengthen the LIBS signal. The choice of a highly polished metallic reflector that suits the used laser wavelength in terms of its reflectivity leads to the back reflection of a considerable portion of the incident laser irradiance. Such reflected laser light reheats the laser-induced plasma on the front surface, strengthening the emission intensity of the spectral lines. Hence, the signal-to-noise ratio experiences an overall improvement that consequently enhances the detection sensitivity of the technique. If the sample is thin enough, all the light will pass through without suffering any perceptible absorption losses; nonetheless, the reflector will reflect more than 97% of the transmitted laser beam. As the sample becomes somewhat thicker, more absorption of the reflected laser light takes place, resulting in a reduction in the enhancement of the intensities of the emitted spectral lines^25^. Therefore, the transmitted and the back-reflected laser light irradiance continues to decrease with increasing the target thickness. The current work presents a comprehensive study on the effect of the experimental parameters, namely the glass target thickness and the laser irradiance, on the signal-to-background ratio SBG in the spectra of the BRELIBS measurements.

## Materials and methods

### Materials

The glass samples used throughout the present study are obtained from the Saint-Gobain glass factory in Egypt. This factory produces the superior annealed glass known as Planilux. During production, the glass is floated, which results in a surface that is as smooth and parallel as possible. Such glass comes in various thicknesses and may be used for many different purposes. The samples used in the present study had three different thicknesses, 1, 3, and 6 mm, with 3 × 3 cm dimensions for each.

### Experimental setup

**Figure 1:**
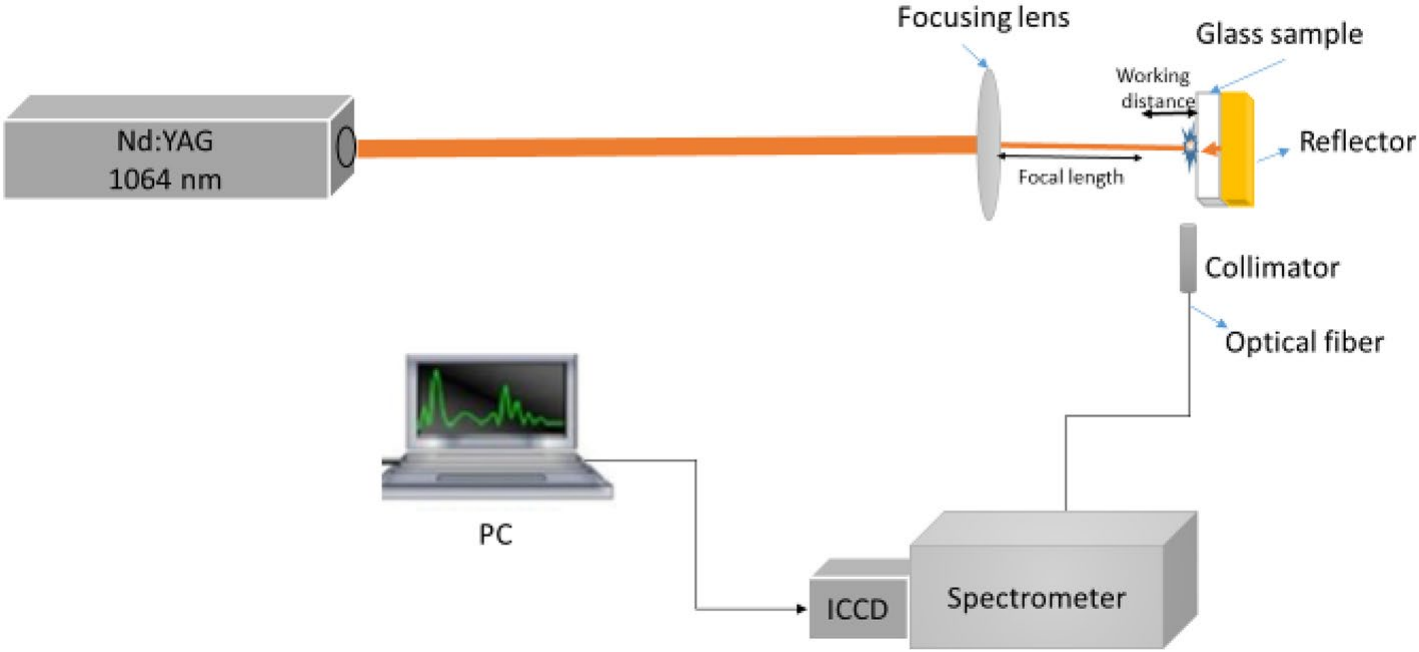
Schematic diagram of the LIBS experimental setup.

The experimental setup used in the present work is composed of a Q-switched Nd: YAG laser (Brio, Quantel, France) at the fundamental wavelength (λ = 1064 nm) with a laser pulse energy of 50 mJ monitored using a Joule meter (SCIENTECH, model AC5001, Boulder, CO, USA). The laser pulse duration was 5 ns (FWHM) at a 20 Hz repetition rate. A planoconvex lens (f = 5 cm) was used to focus the laser beam onto the sample surface with a laser spot size of 26 μm. An XY Z micrometric translational stage was used to mount the sample and adjust the distance between the lens and sample surface to focus precisely on the glass target front surface and to facilitate changing the working distance. A flat metallic reflector (copper or silver) has been highly polished with silicon carbide paper until 1200-grit. Further polishing of the metallic reflectors with diamond pastes has been performed up to ¼ μm to achieve a mirror-like finish and superior surface flatness. The metallic reflector is placed directly on the glass sample’s back surface, as shown in Fig. 1, just above the translational stage. The plasma’s emitted light was collected via a 2 m fused silica optical fiber with a core diameter of 600 μm and fed to the entrance slit of an Echelle spectrometer (Mechelle 7500, multichannel, Sweden). The spectrometer was coupled to an ICCD camera (DiCAM PRO, PCO-Computer optics, Germany) controlled via special multichannel instrument software. The ICCD was triggered optically at a typical delay time of 1500 ns and gate width of 2500 ns for measurements performed in air at atmospheric pressure for all samples. The average of three LIBS spectra was collected per sample; each spectrum represents the sum of 10 laser shots taken at different positions (1 shot at each location on the glass front surface).

Spectra display and analysis were performed using 2D and 3D-Gram/32 software (National Instruments, USA). Moreover, the identification of the spectral lines was carried out via LIBS++ software^26^.

## Results and discussion

### Glass thickness influence

**Figure 2:**
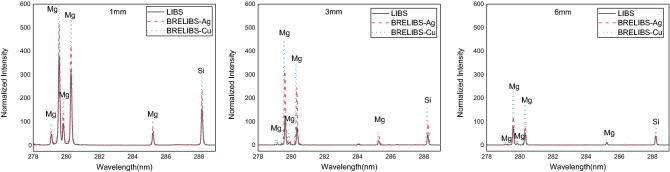
Spectra of LIBS and BRELIBS (with Cu and Ag reflectors) for 1 mm, 3 mm, and 6 mm thick glass samples.

Three transparent glass samples with varying thicknesses were used for this experiment: 1 mm, 3 mm, and 6 mm. The LIBS and BRELIBS spectra for glass with copper or silver reflectors are shown in Fig. 2. Each spectrum is the average of ten single-shot spectra taken at ten different positions on the target surface; every two adjacent spots were 0.5 mm apart. As shown in Fig. 2, a noticeable improvement in the signal-to-background ratio is seen in BRELIBS spectra for the three thicknesses compared to LIBS spectra. These results agree with that of Abdel-Harith et al. for colored glass^24^. Comparing the glass spectra with a copper reflector to the conventional LIBS spectra, the usage of the copper reflector reveals a significant increase in the intensity of the spectral lines. However, the silver reflector enhances spectral lines intensity but less than the copper reflector.

**Figure 3:**
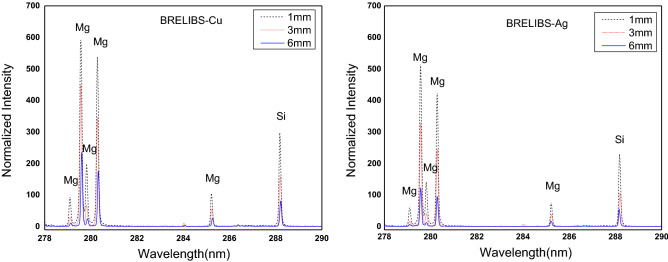
Effect of the sample thickness on the spectral lines intensity of the major element Si and the minor element Mg in the three glass samples of 1, 3, and 6 mm thickness.

**Figure 4:**
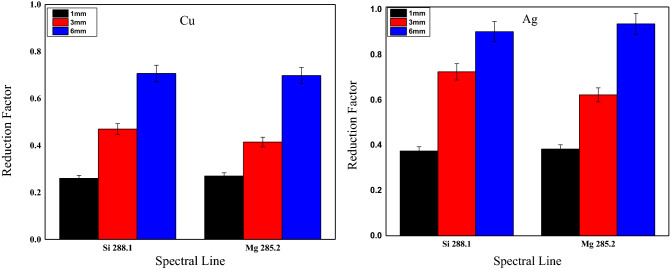
The reduction factor (SBG (LIBS)/SBG (BRELIBS)) for Si and Mg spectral lines for Cu and Ag reflectors.

On the other hand, in terms of thickness effect, Fig. 3 shows the thickness effect on the intensity of the spectral lines of the main element Si and the minor element Mg in the glass target. The depicted spectra demonstrate a significant reduction in the signal-to-background ratio in BRELIBS for both Cu and Ag reflectors behind a glass thickness of 6 mm compared to 3 mm and 1 mm, respectively. The reduction factor (SBG (LIBS)/SBG (BRELIBS)) was calculated and plotted in Fig. 4 to assess the glass thickness effect on the decline in the transmitted and reflected laser light. Two spectral lines of two elements in the glass, i.e., Si (at 288.1 nm) and Mg (285.2 nm) in each glass thickness sample, were used. The reflection-reduction factor is more remarkable in thick glass (6 mm) than in thin ones (3 mm and 1 mm), which could be due to the increase in absorbed energy inside the glass material^27^. The laser pulse energies transmitted through 1 mm, 3 mm, and 6 mm thick glass samples have been measured and listed in Table 1. In its round trip through the glass sample, the laser beam loses more energy in the thicker glass (6 mm).

**Table 1:** The laser pulse energies transmitted through 6 mm, 3 mm, and 1 mm glass samples.

Glass thickness (mm)	Transmitted laser energy (mJ)	Absorbed laser energy (mJ)	Calculated absorbance
1	45.5	4.5	0.04096
3	39	11	0.1079
6	35	15	0.1546

**Figure 5:**
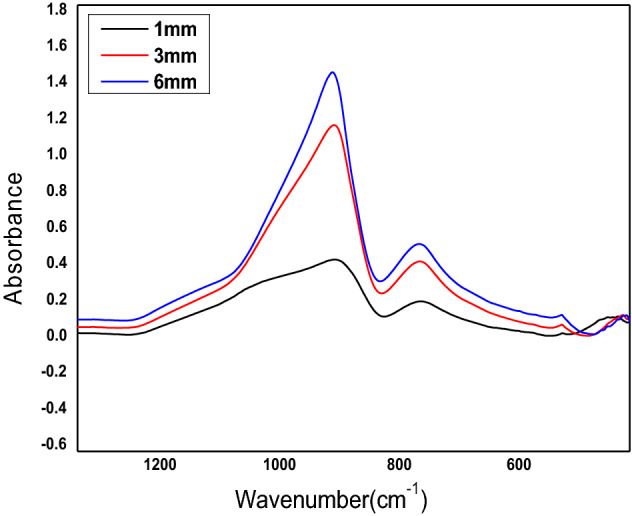
IR absorbance of glass samples, as a function of wave number.

When light travels through a glass, the intensity of the light is typically reduced. This reduction in the light intensity is due to the absorption happening when photons’ energy in the laser beam matches the energy needed to excite electrons within the glass to higher energy states. The absorbance of glass samples, shown in Fig. 5. as a function of wave number, is often used to describe the decrease in light intensity as it travels through the glass. It is defined as^28^

$$A = - log\;\frac{I}{{I_{o} }}$$

where *I*_*o*_ and *I* are the incident and transmitted laser intensities, respectively, the absorbance value depends on the glass’s composition and thickness and the incident light’s wavelength. In this experiment, only glass thickness has been varied, and its effect on the absorbance value is shown in Fig. 5. It has been found that glass thickness of 6 mm has higher absorbance than 3 mm and 1 mm. The calculated values of the absorbance are listed in Table 1.

### Influence of laser irradiance on SBG for LIBS and BRELIBS intensities

The plasma’s emission intensity, temperature, and the charged particles’ density all define the plasma. The impact of adjusting the incident laser irradiance on the spectral line emission of glass samples was investigated at fixed laser parameters (wavelength and pulse duration) and experimental setup. The change in laser irradiance is obtained by changing the working distance (WD), which is the distance between the lens’s focal point and the target surface. i.e., WD = 0 means the sample surface is at the lens’ focal length, moving away from the sample surface (WD > 0) leads to lower irradiance when the beam is defocused, and the laser-focused spot size increases resulting in reduced irradiance. The irradiances corresponding to different WDs are listed in Table 2.

**Table 2:** Laser irradiance for different working distances (WDs).

WD (mm)	Irradiance (GW/cm^2^)
0	1.70 × 10^3^
1	1.63 × 10^3^
2	1.57 × 10^3^
3	1.51 × 10^3^
4	1.46 × 10^3^
5	1.40 × 10^3^

**Figure 6:**
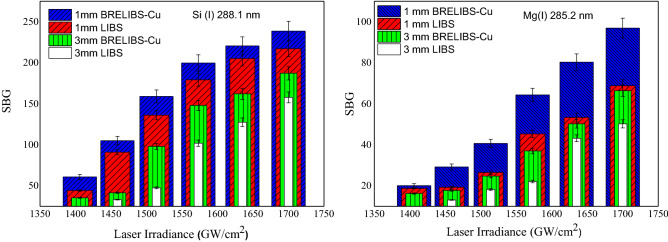
The trends of the SBG ratios of the spectral lines of Si I and Mg I in the LIBS spectra with varying irradiance for two different glass sample thicknesses (1 mm and 3 mm).

A wide range of laser irradiances is used to assess this parameter. For example, it is shown in Fig. 6, the trends of the SBG ratios of the spectral lines of Si I and Mg I in the LIBS spectrum with varying irradiance at atmospheric pressure at two different glass sample thicknesses (1 mm and 3 mm). A pronounced improvement in the SBG ratio in the case of the BRELIBS compared to the LIBS show up for the two different glass thicknesses (1 mm and 3 mm), although the decrease in laser irradiance. On the other hand, the ratio was systematically higher in both cases for the thinner glass. This result is crucial since it demonstrates the possibility of performing BRELIBS analysis even at longer WD up to 5 mm (lower irradiance value) with a reasonable SBG ratio. Mainly, this is because the laser beam reflected from the metallic reflector reheats the plasma plume as it passes through it. Therefore, the specularly reflected laser increases with the increasing of the reflecting area of the metallic reflector due to defocusing. Consequently, the plasma plume reheating increases, and the SBG ratio improves with lower irradiance. For example, there were no measurable spectral lines when using LIBS at WD = 5 mm on a 3 mm thick glass sample. On the contrary, after using the reflectors (BRELIBS), there was an enhancement in the SBG ratio, leading to a well-resolved measurable spectral signal.

### Measurement of electron temperature (T_e_) and electron density (N_e_)

**Figure 7:**
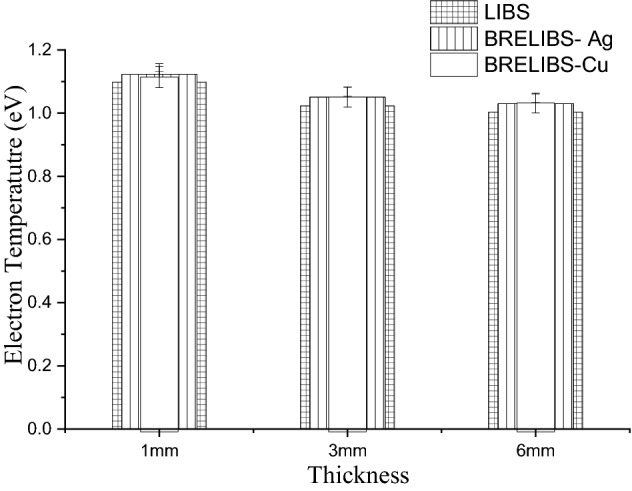
The electron temperature (T_e_) for LIBS, BRELIBS-Ag, and BRELIBS-Cu for the three glass thicknesses.

**Figure 8:**
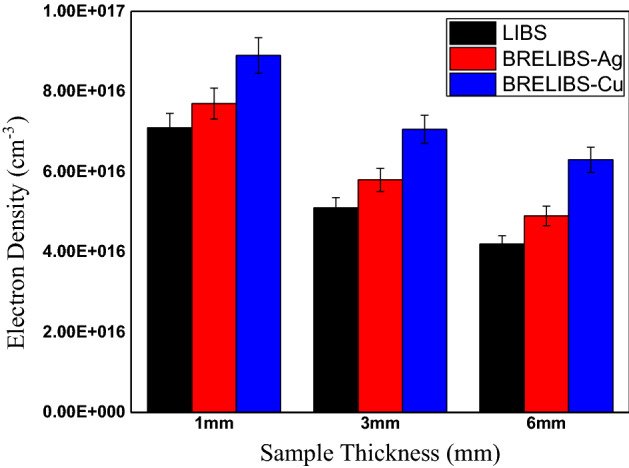
The electron density (N_e_) for LIBS, BRELIBS-Ag, and BRELIBS-Cu for the three glass thicknesses.

As shown above, the glass thickness affects the SBG ratio of LIBS and BRELIBS. Therefore, the plasma parameters, namely electron temperature and electron density, have been estimated and plotted in Figs. 7 and 8, respectively, at the three different glass thicknesses of 1 mm, 3 mm, and 6 mm. The electron temperature T_e_ was obtained using the well-known Boltzmann plot method. As per Abdel-Harith et al., the plasma temperature was determined using the calcium’s atomic spectral lines^24^. The results depicted in the bar graph in Fig. 7 do not reveal any significant differences in the electron temperature for BRELIBS Cu, BRELIBS Ag, and LIBS at the three glass thicknesses.

On the other hand, the value N_e_ was determined using the Stark-broadening of the H_α_ spectral line in the laser-induced plasma emission spectrum of the glass samples with different thicknesses. The electrons’ density in Fig. 8 shows a pronounced increase as the sample thickness decreases. On the other hand, Ne increases slightly in both BRELIBS cases (with Cu and Ag reflectors) compared to those without reflectors (LIBS) in the three glass samples. This higher value of Ne in BRELIBS in the thin glass samples validates the findings about the higher SBG ratio for such samples kind.

## Conclusion

The present study explored the influence of glass thickness and laser irradiance on the SBG ratio in the back-reflection laser-induced breakdown spectroscopy (BRELIBS) spectra. The plasma parameters (electron temperature and electron density) have been measured for different glass thicknesses (1 mm, 3 mm, and 6 mm). As reported before, the results confirmed the merits of using BRELIBS over conventional LIBS by enhancing the SBG ratio. Moreover, the reduction factor, the SBG ratio in LIBS to that in BRELIBS, has a higher value for thicker glass samples than the thin ones for both Cu and Ag reflectors. This was attributed to the increased absorbed laser energy in the thick glass sample than in the thinner one.

Additionally, as the working distances increase, which results in reduced laser irradiance, the SBG ratio of BRELIBS is found to be higher than that of LIBS at the same glass thickness. This result is crucial since it allows conducting BRELIBS measurements even at longer WD up to 5 mm (lower laser irradiance value) with a reasonable SBG ratio. Finally, the comprehensive analysis revealed that the plasma temperature had not been affected much by changing the glass thickness, while the electron density has been slightly increased in the BRELIBS case.

## Data Availability

The datasets used and/or analysed during the current study available from the corresponding author on reasonable request.
